# A study of rural populations’ knowledge, attitude, and practice about brucellosis: a descriptive, cross-sectional, multicenter study

**DOI:** 10.1186/s13104-024-06691-1

**Published:** 2024-01-23

**Authors:** Zahra Montaseri, Zahra Mohebi, Rahil Masoumi, Azizallah Dehghan, Mostafa Bijani

**Affiliations:** 1 Noncommunicable Diseases Research Center (NCDRC), Fasa University of Medical Sciences, Fasa, Iran; 2 Department of Medical Surgical Nursing, School of Nursing, Fasa University of Medical Sciences, Fasa, Iran; 3 Student Research Committee, Fasa University of Medical Sciences, Fasa, Iran

**Keywords:** Brucellosis, Rural populations’, Knowledge, Attitude, Practice

## Abstract

**Objective:**

Brucellosis is a highly contagious disease which is transmitted from animals to humans. One of the populations at high risk of infection is those living in rural areas. The present study was conducted to investigate rural populations’ knowledge, attitude, and practice about brucellosis in Iran. The study used a descriptive, cross-sectional design to assess 300 individuals who were living in rural areas. The subjects were selected using convenience sampling from six villages located in the south of Iran. The data gathered were analyzed using Analysis of variance (ANOVA), and Pearson correlation coefficient in SPSS version 23.

**Results:**

From the 300 individuals who were enrolled in this study, 189 were male and 111 were female. The mean age of the participants was 48.27 ± 4.28 years. The mean scores of the study population’s knowledge, attitude, and practice about brucellosis were found to be low. A significant direct correlation was found between the subjects’ knowledge, attitude, and practice regarding brucellosis.

## Introduction

Brucellosis is one of the most prevalent zoonotic diseases across the world and a major health issue, especially in developing countries [1]. According to World Health Organization (WHO), approximately 500 thousand people are infected with brucellosis every year [2]. In humans, brucellosis can cause external abscesses in different parts of the body; due to the various and lasting complications the infection causes, brucellosis is known as a disease with a thousand faces [3]. Brucellosis is transmitted to human beings via consumption of contaminated milk and dairy products, contact with infected animals and their discharges, meat or marrow through skin, conjunctiva, or lungs. A few cases of transmission through sexual intercourse, placenta, and breastfeeding have also been reported [4]. In general, among Asian and Mediterranean countries, Iran has a wide variety of brucellosis, one reason for which is inadequate control and supervision over imported cattle. Thus, Iran is listed among the countries with a high rate of brucellosis [5]. A good understanding of local knowledge, attitudes, and practices related to brucellosis is necessary for holding an efficient control program, so that information delivery is improved, and relevant control measures are taken.

Brucellosis is an endemic disease in Iran and is mostly prevalent among the villagers and cattlemen who are in direct contact with cattle and dairies at source. Although studies have been conducted in Iran in the field of brucellosis, due to the climate diversity, it is necessary to research the knowledge, attitude, and performance of people in the field of brucellosis in different regions and populations. Investigation of the knowledge, attitude, and performance in the field of brucellosis disease can help the policymakers and managers of the health system to implement control plans and prevent the prevalence of the disease. Therefore, given that no study has been conducted in Fars province in the south of Iran in this field and that there are study gaps that need to be filled, the present study aimed to investigate rural populations’ knowledge, awareness, and practice about brucellosis in Iran in 2023.

## Main text

### Study design

This descriptive cross-sectional multicenter study was conducted from June to September 2022 based on the STROBE Statement (Strengthening the Reporting of Observational Studies in Epidemiology) [6]. The subjects were selected from six villages in Fars Province, south of Iran. The samples were selected using convenience sampling method.

### The inclusion and exclusion criteria

The inclusion criteria were being willing to participate, signing the informed consent form, being literate, being able to communicate, and not having a mental disorder. The subjects who failed to answer over half of the questions on the questionnaires and did not return the questionnaires were excluded.

### Sampling and the study population

The sample size was estimated using the following formula and the findings of a similar study carried out by Nikoonejad et al. [7]. Sample size was set at 257, but, dure to the possibility of attrition, 300 individuals were finally recruited.

$$n = \frac{{{Z^2}.S{D^2}}}{{{d^2}}}$$

In the present study, of the 354 individuals who received the questionnaires, 300 filled them out and returned them, showing a response rate of 85%.

The data collection instrument consisted of two parts. Part one dealt with demographic information with 6 questions on age, gender, education, history of infection with brucellosis, contact with cattle, and dairy consumption. Part two was a knowledge, attitude, and practice (KAP) questionnaire developed and evaluated by Mligo et al. in 2022. In this questionnaire, the knowledge questions consist of 17items which are scored using a 5-point Likert scale (completely agree = 5 and completely disagree = 1). A score between 1 and 35 indicates poor, knowledge a score between 36 and 70 indicates average knowledge, and a score between 71 and 85 indicates satisfactory knowledge. The attitude questions consist of 9 items scored on a 5-point Likert scale, ranging from completely agree = 5 to completely disagree = 1. A score between 1 and 25 indicates poor, attitude a score between 26 and 35 indicates average attitude, and a score between 36 and 45 indicates satisfactory attitude. The practice section of the questionnaire consists of 10 questions which are scored on a 3-point scale (never = 0, rarely = 1, and frequently = 2). The total score for this part is 20. A score between 0 and 10 indicates poor, practice a score between 11 and 15 indicates average practice, and a score between 16 and 20 indicates satisfactory practice. The reliability of this questionnaire was approved with a Cronbach’s alpha of 0.95 in Mligo’s study [8].

### Translation procedure

After we obtained written permission from the designer of the scale, we started the translation process [9]. To do so, we used forward-backward method of translation. First, two translators who were fluent in the Persian and English languages translated the questionnaire into Persian. Then, we checked the two translations to select the best one for each item. Then, the designed Persian questionnaire was translated into English by two translators who were not familiar with the original questionnaire. After we checked, revised, corrected, and merged these two translations, we sent it to the designer of the questionnaire. Then, the items were approved as to their similar meanings.

Content validity was used to determine the validity of the questionnaire. To measure content validity, we determined the Content Validity Ratio (CVR) and Content Validity Index (CVI) of the questionnaire. As to CVR, a panel of experts assessed the necessity of the items and rated them as ‘necessary’, ‘useful but not necessary’, and ‘not necessary’. Accordingly, 15 experts were consulted and the items with values higher than 0.49 were considered as acceptable based on the Lawshe Table [10]. The CVRs of 36 items were found to be higher than 0.49. As to CVI, the 15 experts were asked to rank the items in terms of relevance, clarity, and simplicity; scores higher than 0.79 were considered as acceptable. The results revealed that the CVIs of 36 items were higher than 0.79. The reliability of the instrument was determined using the test-retest method. Accordingly, the questionnaire was completed by 100 individuals who lived in rural areas in Fars Province in the south of Iran in two stages with a two-week interval. The intra-class correlation coefficient (ICC) for the 36 items was 0.79, indicating that the questionnaire had appropriate internal consistency.

### Data analysis

The data were analyzed using descriptive statistics which included mean and standard deviation and inferential statistics consisting of Analysis of variance (ANOVA), and Pearson correlation coefficient in SPSS version 23.

## Results

189 out of the 300 individuals who participated in the study were male and 111 were female. Table 1 displays the participants’ demographic features. Table 2 the results revealed that the mean and standard deviation of the subjects’ scores for knowledge, attitude, and practice regarding brucellosis were at a low level. Also, it was shown that more than half of the subjects (57.3%) had a history of infection with brucellosis. Moreover, more than half of the subjects were involved in buying and selling cattle, milk, and dairy products; 70% of them did not boil milk before selling it; and 39% had seen their cattle miscarry, which is a symptom of brucellosis in the cattle. In the present study, the places where dairy products had been sold were examined (Table 3). Approximately half of the dairy products were sold to dairy companies, and 52% were directly sold to end consumers. The correlation between the subjects’ gender on the one hand and their knowledge, attitude, and practice on the other was found to be insignificant. However, as shown in Table 4, the correlation between the subjects’ history of infection with brucellosis on the one hand and their knowledge, attitude, and practice on the other was found to be significant (*P* < 0.001). Moreover, a significant correlation was observed between the places where milk was sold and the subjects’ knowledge, attitude, and practice regarding brucellosis (*P* < 0.001) (Table 5).

**Table 1:** Frequency distribution of the subjects in terms of education, gender, knowledge, attitude, and practice

Variable		Frequency	Percentage
Gender	Male	189	63
	Female	111	37
Education	Uneducated	50	16.7
	Elementary school	45	15
	Junior high	79	26.3
	High school	82	27.3
	College degree	44	14.7
Knowledge	Poor	154	51.33
	Average	110	36.66
	Satisfactory	36	12
Attitude	Poor	148	49.33
	Average	120	40
	Satisfactory	32	10.66
Practice	Poor	164	54.66
	Average	121	40.33
	Satisfactory	15	5

**Table 2:** The subjects’ brucellosis knowledge, attitude and practice test scores

Variable	Number	Min.	Max.	Mean	SD
Knowledge	300	16	32	21.2700	2.50
Attitude	300	5	12	12.8900	1.14
Practice	300	8	22	9.5900	3.67

**Table 3:** Frequency distribution of the subjects’ exposure to risk factors

Variable		Frequency	Percentage
History of infection with brucellosis	Yes	172	57.3
	No	128	42.7
Buying and selling cattle frequently	Yes	159	53
	No	141	47
Buying and selling dairies frequently	Yes	226	75.3
	No	74	24.7
Boiling milk before selling it	Yes	88	29.3
	No	212	70.7
Selling cattle in the past year	Yes	113	37.7
	No	187	62.3
History of miscarriage in their cattle	Yes	117	39
	No	183	61

**Table 4:** The correlation between the subjects’ brucellosis knowledge, attitude and practice and their history of infection with brucellosis

Variable	History of infection with brucellosis	Number	Mean	SD	*P*-value*
Knowledge	Yes	172	21.0872	2.10220	0.006
	No	128	21.5156	1.95663	
Attitude	Yes	172	9.8547	1.02986	0.047
	No	128	9.9375	1.29657	
Practice	Yes	172	12.2035	3.51972	0.029
	No	128	13.1094	3.82539	

Pearson correlation coefficient

**Table 5:** The correlation between the subjects’ brucellosis knowledge, attitude and practice and the point of selling dairies

knowledge, attitude and practice		Number	Mean	SD	SE	Confidence interval		*P*-value*
						Lower bound	Higher bound	
Knowledge	Local markets	111	20.6667	1.92754	0.18954	20.2910	21.0423	0.025
	Neighboring villages	43	21.6279	1.21499	0.29395	21.0347	22.2211	
	Relatives, friends, neighbors	7	22.1429	2.01561	0.45922	21.0192	23.2665	
	Fellow villagers	27	21.2963	1.07415	0.38790	20.4989	22.0936	
Attitude	Local markets	111	10.0270	0.84581	0.10195	9.8250	10.2291	0.000
	Neighboring villages	43	9.3721	0.53452	0.12898	9.1118	9.6324	
	Relatives, friends, neighbors	7	9.5714	0.65590	0.20203	9.0771	10.0658	
	Fellow villagers	27	10.7407	3.89445	0.12623	10.4813	11.0002	
Practice	Local markets	111	12.2793	2.63758	0.36964	11.5467	13.0118	0.006
	Neighboring villages	43	11.7442	1.67616	0.40223	10.9325	12.5559	
	Relatives, friends, neighbors	7	12.1429	4.52281	0.63353	10.5927	13.6930	
	Fellow villagers	27	14.9259	1.92754	0.87042	13.1368	16.7151	

Analysis of variance (ANOVA)

## Discussion

In the current study, the knowledge, attitude, and practice of 300 villagers in Fars Province, south of Iran as to brucellosis were evaluated. It was found that their knowledge, attitude, and practice as to brucellosis were at a low level. In a study carried out by Mligo et al., the knowledge, practice, and attitude of the subjects toward brucellosis was at an average level [8]. A study by Madut et al. (2017) in Sudan showed that farmers’ and cattlemen’s knowledge and practice regarding brucellosis and ways of preventing it were poor [11], Likewise, Lindahl et al. (2017) demonstrated that the villagers in Tajikistan had poor knowledge, attitude and practice regarding brucellosis and ways of preventing it [12].

In the present study, the subjects’ history of contact with cattle and history of infection with brucellosis were addressed as well. More than half of the subjects (57.3%) surveyed had a history of infection with brucellosis. In view of the high percentage of the subjects who had contracted the infection, the role of knowledge, attitude, and practice regarding brucellosis in this population can be enlightening. In a study conducted in Yazd, (2007), located in the southeast of Iran, 75% of the infected subjects had been living in rural areas [13]. These findings show that brucellosis is an endemic disease in Iran. In the present study, 226 subjects were directly engaged in buying and selling milk, yogurt, and other dairy products. In other words, more than two thirds of the study population were in contact with the dairies they consumed or distributed at source. In addition, less than one third of the subjects (88 individuals) were found to boil milk before selling it. Similarly, in a study carried out by Lindahl et al. (2017) in Tajikistan, about 70% of the subjects sold their dairy products without pasteurizing them first [12]. According to a study by Nikoonejad et al. (2020), approximately 70% of the rural population believe that boiling milk will reduce its nutritional value [7].

The food culture of people residing in villages and the increasing price of meat and dairies have resulted in disregarding the principles of disposal of contaminated cattle; this has led to an increase in the rate of brucellosis in humans. In the same vein, other studies [14–15] have reported that brucella is transferred by consumption of unpasteurized dairy products. Moosazadeh et al. (2016) revealed that 57.1% of the patients studied contracted brucellosis through consumption of contaminated and unpasteurized dairy products [14]. In the present study, more than half of the subjects had a history of direct contact with cattle or were regularly involved in buying and selling cattle. According to a meta-analysis conducted by Moosazadeh et al. (2016), many brucellosis patients were infected as the result of direct contact with contaminated cattle [14]. These findings emphasize the significance of educating villagers about the precautions they should take in handling cattle and their discharges and vaccinating all their cattle. However, villagers are not very interested in learning about brucellosis, and it is possible that cattlemen do not receive enough education about this disease. Studies by Esmaili [15] and Babaei et al. [16] highlight the importance of educating cattlemen about high-risk behaviors as to brucellosis. The findings of the present study indicated that the subjects’ knowledge was poor, but they had a poor attitude to brucellosis, the issue which should be addressed in farmers and cattlemen.

### Limitations

The study was conducted in one province in the south of Iran, so the findings may not be generalizable to other parts of the country. Thus, it is recommended that similar studies should be conducted in other regions of Iran and other countries.

## Conclusion

In the present study, the knowledge, attitude, and practice of the rural population regarding brucellosis were found to be poor. It is suggested that the healthcare policymakers should take effective measures to educate rural populations about the methods of preventing brucellosis, so that the spread of the infection could be controlled.

## Data Availability

The datasets generated and/or analysed during the current study are not publicly available due to the necessity to ensure participant confidentiality policies and laws of the country but are available from the corresponding author on reasonable request.
